# Strengthening human resources for health systems resilience to care for mothers and children

**DOI:** 10.1186/1472-6963-15-S1-S6

**Published:** 2015-06-08

**Authors:** Uta Lehmann

**Affiliations:** 1 School of Public Health, University of the Western Cape, South Africa

## 

What do we need to do better to ensure better health, and less preventable morbidity and mortality among mothers, babies and young children? This is the question asked by the papers of this special edition. In their introductory commentary, Fotso and Fogarty point to substantial progress made in improving young children’s health, and some progress in improving health for mothers. But clearly our efforts globally are not good enough if eight million children and 350,000 pregnant and postpartum women still die annually of preventable causes. The answer is simple and yet target-defeatingly complex: access to skilled, equipped, supported and motivated health workers who serve women through pregnancy, delivery and postpartum care. Furthermore, both question and answer are ultimately not about MNCH, nor ‘just’ about human resources for MNCH, but about functioning and resilient health systems, which provide the frame, the resources, and the support within which health workers function and interact with communities and young mothers.

The *Joint Learning Initiative* and the 2006 *World Health Report* gave us a rough estimate of what this might mean at a minimum: 2.3 health workers per 1,000 population to achieve skilled attendance at birth [1,2]. Yet even on this minimum target we are far behind: in low income countries, fewer than 50% of deliveries are attended by a skilled birth attendant (http://www.who.int/gho/maternal_health/en/). A recent analysis conducted by the Global Health Workforce Alliance and World Health Organization (WHO) estimated a gap of 7.2 million professional health workers in 2012, a number that is set to rise to 12.9 million over the next decades [3].

Numbers are not the only problem. Access is a multi-faceted challenge, requiring attention to health systems ‘hardware’: availability (what cadres? with what skills? where? when?) and logistics (transport, funding, equipment, drugs). It also requires attention to health systems ‘software’: attitudes (values, beliefs, motivation of health workers as well as community members), and relationships (between health workers and between health workers and communities) [4].

The papers in this collection address ‘software’ challenges: interventions which explore how to better care for carers, as well as interventions which aim to strengthen relationships between professionals from the formal health system and health care workers beyond the formal system: community volunteers, traditional birth attendants and even husbands accompanying their wives to health facilities and assisting with delivery support. All but one of them explore these themes in contexts of heightened fragility, as countries recover from civil war, a theme that is all too common across Africa. These themes are also explored in other low- and middle-income countries, and are increasingly being highlighted HRH debates globally [5-9].

All papers address health systems resilience: resilience within and between people. Both papers focusing on Sierra Leone discuss interventions which aimed to strengthen health workers’ sense of self, in one case through teaching coping strategies (Helping Health Workers Cope project), in the other through peer learning using the Quality Circles approach. In both cases, internal well-being led to a greater ability to ‘take perspective’ and recognize others’ roles and strengths, thus reconstructing collaborative work and networks.

While the interventions reported in both of these papers have a strong focus on repairing damage caused not only by chronic shortages, but more acutely many years of civil war, the Weiss et al study set in Burundi raises the important issue of ensuring sustainability of care programs by ’mainstreaming’ support and supervision of volunteers in the health care system. Evidence internationally shows that Community Health Worker projects relying on non-governmental organizations for resources and support remain add-ons and thus by definition are fragile [10-12]. Exploring the feasibility of building supervision and support into the scope of work of the formal system is thus an important step in strengthening sustainable community-based care. The study also highlights the challenge of mainstreaming such support under conditions of critical human resources shortages in the formal sector, as additional functions are not easily allocated. Whether relatively underutilized cadres of health workers (junior nurses) could effectively and sustainably take on support functions deserves further exploration.

The last paper by Fotso et al on male engagement in delivery of MNCH explores largely uncharted terrain of building resilience by extending the pool of people able to actively support access to maternal and newborn health services. The effort to reach husbands and fathers through extending the well-established Accredited Social Health Activists program in India to include Male Health Activists introduces innovative thinking, such as involving men to accompany woman in labor to facilities at night, thereby overcoming very practical health care challenges. Their involvement no doubt also facilitates buy-in and understanding of community-based health services, thus creating additional layers of resilience at the interface between the formal health system and communities.

One way of thinking about the four interventions presented here is as projects along a continuum of program strengthening and systems resilience: resilience as repairing damage caused in the past; resilience as strengthening systems going forward; and resilience as introducing new actors and extending traditionally female-dominated community-level care to include men.

The importance of such resilience is presently being highlighted in the battle to conquer the Ebola epidemic in West Africa. The World Health Organization and other actors have acknowledged that “Ebola became epidemic in Guinea, Liberia, and Sierra Leone in large part because of their weak health systems” and that “countries need to create resilient integrated systems that can be responsive and proactive to any future threat.” (Marie-Paule Kieney [13])

The interventions discussed in this special edition point to leverage points at the core of building resilient health systems through strengthening human resources. In moving forward, the task for practitioners and researchers lies is to test such small scale interventions over time and at scale: who would train and support Community Health Workers and their routinely in the Burundian health system? Are there in fact substantial numbers of underutilized staff in the system? How could Helping Health Workers Cope and Quality Circle interventions in Sierra Leone be built into the foundation of the health system? Is it feasible to employ Male Health Activists on a large scale and will men remain engaged over long periods of time?

Finding answers to these questions will ultimately strengthen human resources and build health systems, which will not only contribute to the reduction of preventable deaths, but will also ensure the health of mothers and children globally.

## Competing interests

The author declares no competing interests.

## Declarations

The publication costs for this article were funded by the author.

This article has been published as part of *BMC Health Services Research* Volume 15 Supplement 1, 2015: Improved access to maternal, newborn and child health services: strengthening human resources for health. The full contents of the supplement are available online at http://www.biomedcentral.com/bmchealthservres/supplements/15/S1.
